# Medication Errors in Primary Healthcare: A Systematic Review of Their Causes, Prevention, and Impact on Patient Safety

**DOI:** 10.7759/cureus.112589

**Published:** 2026-07-13

**Authors:** Elham Qanadeely, Fawziah Roublah

**Affiliations:** 1 Preventive Medicine, Saudi Commission for Health Specialities, Makkah, SAU; 2 Orthopaedic Surgery, Faculty of Medicine, King Abdulaziz University, Jeddah, SAU

**Keywords:** adverse drug reactions, interventions, medication errors, medication safety, patient safety, primary care

## Abstract

Medication errors and adverse drug reactions (ADRs) remain major patient safety concerns in primary healthcare settings worldwide, contributing substantially to preventable morbidity, mortality, and healthcare costs. This systematic review synthesized evidence regarding the prevalence, causes, prevention strategies, and impacts of medication errors in primary care. A comprehensive search of PubMed, EMBASE, CINAHL, Cochrane Library, and Web of Science was conducted for studies published between 2013 and 2024. Eligible studies included randomized controlled trials, observational studies, qualitative studies, and systematic reviews with meta-analyses focusing on medication safety in primary healthcare settings. Thirteen studies involving more than 1.7 million participants were included. Medication errors were highly prevalent, particularly among older adults, patients with chronic diseases, and individuals exposed to polypharmacy. Common medication-related problems included inappropriate dosing, prescribing errors, ADRs, and drug interactions. Communication failures, insufficient medication reconciliation, lack of provider training, and systemic healthcare barriers were frequently identified contributing factors. Preventable adverse drug events were associated with significant clinical and economic burdens, including increased hospitalizations and healthcare costs. While professional and organizational interventions demonstrated variable effectiveness, pharmacist-led medication reviews and clinical pharmacy services consistently improved medication safety outcomes and reduced prescription-related costs. However, the certainty of this evidence is limited by heterogeneity in study designs, settings, and outcome definitions and the moderate methodological quality of several included studies. The findings emphasize that medication errors in primary care remain a significant global health challenge requiring multifaceted interventions focused on communication, education, standardized protocols, clinical pharmacy integration, and patient-centered approaches to improve medication safety and healthcare quality.

## Introduction and background

Medication errors represent a significant challenge within the healthcare system, particularly in primary care settings. These errors primarily involve incorrect prescriptions, dosages, drug interactions, and allergies, and it is believed to result from communication failures among healthcare teams and issues throughout the medication chain [1,2]. The World Health Organization (WHO) has highlighted that medication errors are not only common but also potentially preventable, with estimates suggesting that they contribute to a substantial number of adverse drug events (ADEs) globally. In primary care, where the majority of prescriptions are issued, the prevalence of these errors is particularly concerning, resulting in a median prevalence rate of 12.8% ADEs [3].

A medication error is any preventable event that may cause or lead to inappropriate medication use or patient harm while the medication is in the control of a healthcare professional, patient, or consumer. An ADE is any injury resulting from an intervention related to a drug and may or may not be preventable. An adverse drug reaction (ADR) is a harmful or unintended response to a medicinal product used at normal doses. A prescribing error is a subtype of medication error arising at the prescribing stage, such as incorrect drug selection, dose, frequency, or duration [1,2].

The complexity of medication management in primary care is exacerbated by an aging population with multifaceted health needs and the increasing introduction of new medications [4]. This demographic shift necessitates careful consideration of prescribing practices to avoid errors that can lead to serious health consequences, including hospitalization and even death [4]. The types of medication errors commonly reported include incorrect dosages, inappropriate drug selections, and failures to account for patient-specific factors such as allergies and comorbidities [5]. 

Understanding the causes of medication errors is essential for developing effective prevention strategies. Factors contributing to these errors can be categorized into several domains: healthcare professional-related factors (such as inadequate training or knowledge), patient-related factors (including literacy and understanding of medications), environmental factors (like workload and distractions), and systemic issues (such as poor communication among healthcare providers) [1,2]. Each of these domains plays a crucial role in the occurrence of medication errors and highlights the need for comprehensive approaches to enhance patient safety.

Preventing medication errors requires multifaceted interventions tailored to address the specific challenges faced in primary care. Strategies may include implementing standardized protocols for prescribing and dispensing medications, enhancing communication among healthcare teams, utilizing health information technology to support decision-making, and fostering a culture of safety that encourages reporting and learning from errors [6,7]. Additionally, involving pharmacists in the medication management process has been shown to reduce error rates significantly [7]. Moreover, implementing information technology, enhancing drug labeling, and medication reconciliation can improve medication safety [8]. Establishing a medication safety culture and promoting collaboration among healthcare professionals are also crucial for minimizing errors [2]. Despite these efforts, medication errors remain a leading cause of patient harm in primary care [9,10], necessitating continued research and intervention development.

The impact of medication errors on patient safety cannot be overstated. ADEs resulting from these errors can lead to increased healthcare costs, prolonged hospital stays, and diminished quality of life for patients [11]. Moreover, the emotional toll on both patients and healthcare providers can be profound. Beyond their direct clinical consequences, medication errors undermine patient trust and impose substantial economic costs on health systems. Errors can also increase the risk of drug-drug interactions, and healthcare professionals involved in errors may experience considerable distress, a phenomenon described as “second victim syndrome” [12]. Medication errors may also carry medicolegal consequences for healthcare providers and institutions [13]. Therefore, it is crucial to address medication errors to ensure patient safety and healthcare quality.

Although previous systematic reviews have examined medication errors in outpatient and ambulatory settings, most recently that by Naseralallah et al. [5], which focused principally on prevalence and contributing factors, few have integrated evidence on prevalence, causes, clinical and economic impact, and the effectiveness of prevention strategies specifically within primary healthcare. To address this gap, this systematic review aims to synthesize existing literature on the causes, prevention strategies, and impacts of medication errors in primary healthcare settings. By identifying key themes and gaps in current knowledge, this review seeks to inform future research directions and practical interventions aimed at improving patient safety outcomes. Enhancing our understanding of medication errors will contribute to safer healthcare practices and better health outcomes for patients across diverse populations.

## Review

Methods

Study Design

This systematic review was designed to synthesize evidence on the prevalence, causes, impact, and prevention of medication errors in primary care settings. To ensure methodological rigor and transparency, the review adhered to the Preferred Reporting Items for Systematic Reviews and Meta-Analyses (PRISMA) guidelines [14], which were instrumental in structuring the review process and facilitating reproducibility. The review protocol was not prospectively registered in PROSPERO or any other registry; this is acknowledged as a limitation.

Eligibility Criteria

The review included studies that met specific inclusion criteria. Eligible studies focused on patients and healthcare providers in primary care settings, such as general practice, outpatient care, and community-based healthcare. Studies addressing medication errors, ADEs, and their prevention strategies were prioritized. Outcomes of interest included the prevalence and incidence of medication errors, their causes, impacts, and the effectiveness of prevention interventions. Observational studies (cross-sectional, cohort, and case-control), qualitative studies, randomized controlled trials (RCTs), and systematic reviews with meta-analyses containing extractable primary quantitative data were included, provided they were published in English between January 2013 and December 2024 to account for the latest updated knowledge in the literature. Narrative reviews, editorials, opinion papers, conference abstracts, institutional reports, and dissertations were excluded. Studies conducted in mixed or adjacent settings (e.g., national surveillance networks, home care, or studies spanning both ambulatory and hospital care) were eligible only where they reported data relevant to medication safety in primary or community-based care; this pragmatic approach was adopted to capture the full continuum of community medication management, and its implications are addressed in the limitations. Because both primary studies and systematic reviews were eligible, evidence from these two sources was synthesized and reported separately; no quantitative pooling across study types was performed, and the reference lists of included systematic reviews were cross-checked against the included primary studies to identify potential overlap and minimize double-counting of participants. 

Data Sources and Search Strategy

A comprehensive literature search was conducted using multiple electronic databases, including PubMed, EMBASE, CINAHL, Cochrane Library, and Web of Science. The search strategy incorporated Medical Subject Headings (MeSH) terms and keywords and followed the Population, Intervention, Comparison and Outcome (PICO) framework as shown in Tables 1, 2. The final search of all databases was conducted in March 2026. Searches were restricted to English-language publications dated January 2013 to December 2024; no other filters or truncations were applied. The complete database-specific search strings, including Boolean operators, are reported in Table 2. The full search strategy is also reported in the Appendices.

**Table 1:** PICO framework

Component	Description
Population (P)	Patients and healthcare providers in primary care, outpatient clinics, general practice, ambulatory care, and community healthcare settings
Intervention/Exposure (I)	Medication safety interventions, medication reviews, clinical pharmacy services, prescribing systems, reconciliation processes
Comparator (C)	Standard care, absence of intervention, or comparison between intervention types
Outcomes (O)	Medication errors, adverse drug events, adverse drug reactions, prescribing errors, preventability, patient safety outcomes, healthcare costs

**Table 2:** Search strategy across databases

Database	Search Strategy
PubMed	(“medication errors”[MeSH] OR “adverse drug events” OR “adverse drug reactions”) AND (“primary care” OR “general practice” OR “ambulatory care”) AND (“prevalence” OR “causes” OR “prevention” OR “impact”)
EMBASE	(‘medication error’/exp OR ‘adverse drug event’) AND (‘primary care’ OR ‘general practice’) AND (‘prevention’ OR ‘patient safety’)
CINAHL	(“medication errors” OR “drug-related problems”) AND (“primary healthcare”) AND (“patient safety” OR “prevention strategies”)
Cochrane Library	(“medication errors” AND “primary care”)
Web of Science	TS=(“medication errors” OR “adverse drug events”) AND TS=(“primary care” OR “general practice”)

Study Selection

The study selection process was conducted in two stages. First, two reviewers independently screened the titles and abstracts of all retrieved articles to exclude those irrelevant to the review’s objectives. In the second stage, full-text articles of potentially eligible studies were reviewed to confirm their inclusion based on the eligibility criteria. Discrepancies between reviewers were resolved through discussion or consultation with a third reviewer, ensuring a robust and unbiased selection process. The entire selection process was documented using a PRISMA flow diagram. All disagreements among reviewers were resolved by consensus.

Data Extraction

Data were extracted from the included studies using a standardized data extraction form. The form captured key study characteristics such as author(s), publication year, study design, sample, and main findings, encompassing prevalence/incidence rates, causes and impacts of medication errors, and details of prevention strategies. Two reviewers independently conducted data extraction to ensure accuracy and consistency.

Quality Assessment

For observational studies assessed using the Newcastle-Ottawa Scale (NOS) [15], studies scoring 7-9 points were categorized as high quality, 4-6 points as moderate quality, and 0-3 points as low quality. For RCTs assessed using the Cochrane Risk of Bias Tool [16], studies with low risk across most domains were considered high quality, those with some concerns were considered moderate quality, and studies with high risk of bias across multiple domains were categorized as low quality. Systematic reviews assessed using AMSTAR-2 [17] were classified according to critical methodological weaknesses and overall confidence ratings. Summary quality categories are presented in the quality assessment table, and detailed domain-level assessments for each included study are provided in the Appendices.

Data Synthesis

A narrative synthesis approach was employed to analyze and present the findings due to the heterogeneity of study designs, populations, and outcome measures. Results were organized into thematic areas corresponding to the study objectives: prevalence and incidence of medication errors, causes, impacts (both clinical and economic), and prevention strategies. For studies reporting numerical data, descriptive statistics such as means, medians, and ranges were used to summarize findings. Studies were grouped for synthesis by outcome domain and, within each domain, by study design (quantitative primary studies, qualitative studies, and evidence syntheses), and outcomes were categorized, with medication errors, adverse drug events, and adverse drug reactions reported separately wherever the primary studies allowed. A meta-analysis was not performed because the included studies differed substantially in design, populations, settings, outcome definitions, and measurement methods; this clinical and methodological heterogeneity meant that statistically comparable effect estimates could not be derived.

Ethical Considerations

This systematic review analyzed data from previously published studies and did not involve human or animal participants directly. Therefore, ethical approval was not required. 

Results

Included Studies and Their Main Characteristics

**Figure 1:**
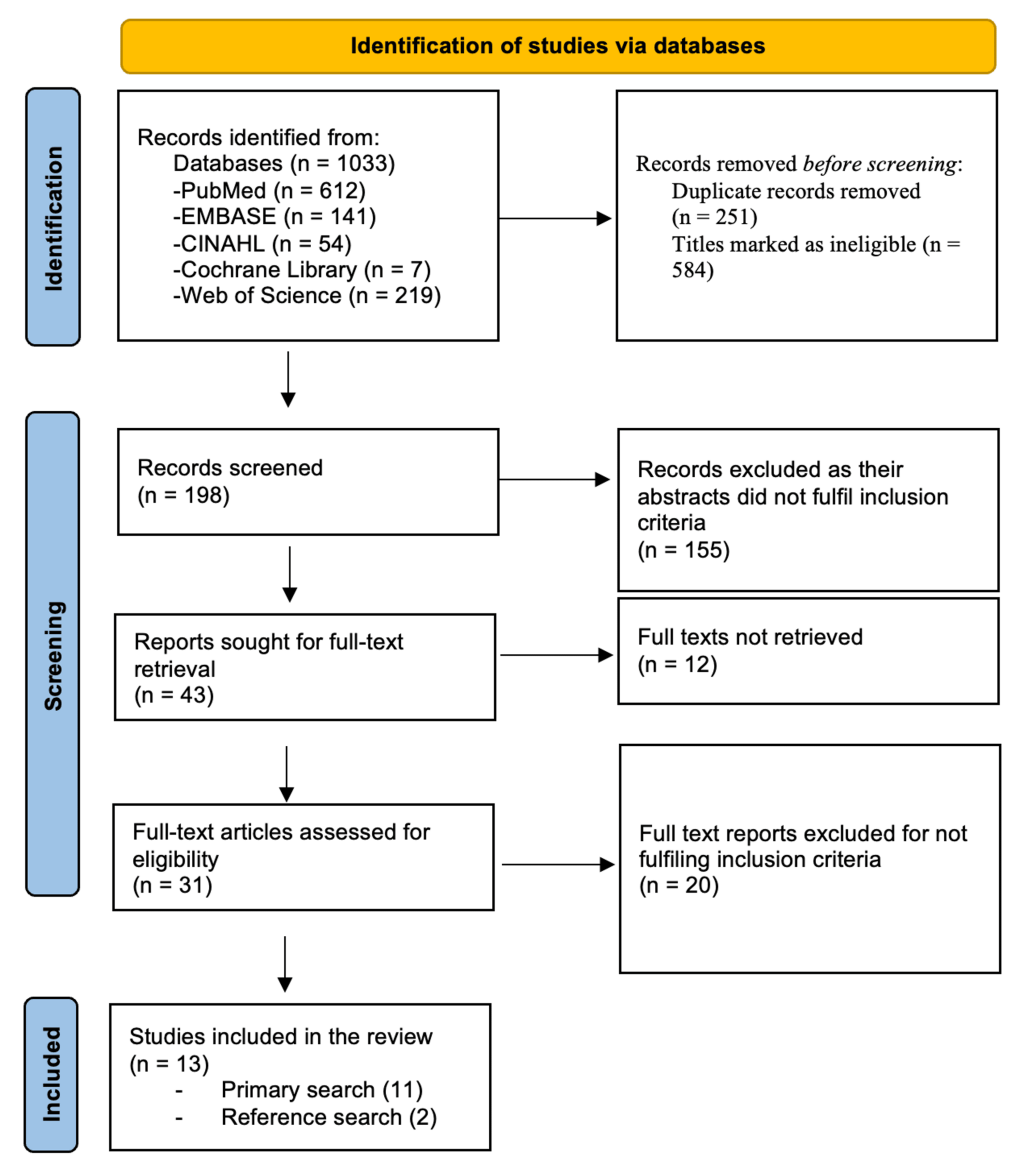
PRISMA flow chart illustrating the study selection process PRISMA: Preferred Reporting Items for Systematic Reviews and Meta-Analyses

The search of electronic databases initially yielded 1033 items in total. After removal of 251 duplicate records and exclusion of 584 records at the title-screening stage, 198 records remained for abstract screening. Of these, 155 were excluded because their abstracts did not fulfil the inclusion criteria, leaving 43 reports for which full-text retrieval was attempted. Twelve full texts could not be retrieved, so 31 full-text articles were assessed for eligibility. Following full-text screening, 20 articles were excluded for not fulfilling the inclusion criteria, yielding 11 eligible studies from the database search. Screening of the reference lists of relevant articles identified two additional eligible studies, giving a final total of 13 included studies (Figure 1).

The included studies are diverse in terms of sample size, design, and focus. They include RCTs, observational studies, qualitative studies, systematic reviews and meta-analyses, and mixed-methods studies. The study designs range from small qualitative studies with 10-26 participants to large-scale systematic reviews involving over 1.5 million participants. The focus areas include medication errors, ADRs, barriers to medication safety, and intervention effectiveness (Table 1). Studies were conducted in various healthcare settings, including primary care clinics, hospitals, home care environments, and national surveillance networks. The populations studied included general populations and specific groups, such as older adults, polypharmacy patients, and those with mental illness. The geographical scope of the studies included resource-rich countries like England and Switzerland, as well as resource-limited contexts.

**Table 3:** Characteristics of the included studies *:* *Year of publication; RCTs: randomized controlled trials

No.	Title	Authors/Year*	Sample	Study Design	Main Findings
1	Professional, structural, and organizational interventions in primary care for reducing medication errors.	Khalil et al. [18] 2017	169,969 participants across 30 studies	Systematic review of RCTs	Professional and organizational interventions showed limited or uncertain effectiveness in reducing hospital admissions, emergency visits, or mortality related to medication errors.
2	PRIME study: Prescription review to impede medication errors.	Adil et al. [19] 2020	1,149 participants	Prospective observational study	Prescription errors accounted for most medication errors (87.1%). Medication reviews significantly reduced the average number of errors per patient during follow-up.
3	Economic analysis of the prevalence and clinical and economic burden of medication error in England	Elliot et al. [20] 2021	Not applicable	Retrospective observational analysis	Medication errors were estimated at 237 million annually in England and were associated with major economic costs, hospital bed use, and mortality.
4	Medication errors in home care: a qualitative focus group study	Berland et al. [21] 2017	20 registered nurses	Qualitative focus group study	Communication failures, inadequate information exchange, and insufficient staff competence were major contributors to medication errors in home care settings.
5	Prevalence of adverse drug reactions in the primary care setting: A systematic review and meta-analysis	Insani et al. [22] 2021	1,568,164 participants	Systematic review and meta-analysis	Adverse drug reactions were common in primary care, with approximately one-fifth considered preventable. Cardiovascular medications were most frequently implicated.
6	Sources of unsafe primary care for older adults: a mixed-methods analysis of patient safety incident reports	Cooper et al. [23] 2017	1,591 incident reports	Cross-sectional mixed-methods study	Medication-related incidents, communication failures, and poor clinical decision-making were the leading contributors to unsafe care among older adults.
7	Medication safety challenges in primary care: Nurses’ perspective	Khalil et al. [24] 2018	10 participants	Qualitative interview-based study	Barriers to medication safety included unclear reporting systems, insufficient education, and lack of clarity regarding nurses’ responsibilities in medication management.
8	Frequency of medication errors in primary care patients with polypharmacy.	Koper et al. [25] 2013	169 participants	Cross-sectional observational study	Polypharmacy was strongly associated with dosing errors, inappropriate prescriptions, and potentially harmful drug interactions, especially among older adults.
9	Medication incidents in primary care medicine: a prospective study in the Swiss Sentinel Surveillance Network (Sentinella)	Gnädinger et al. [26] 2017	167 participants	Prospective observational surveillance study	Inappropriate dosing and prescribing the wrong medication were the most frequent medication incidents identified in primary care practice.
10	Incidence and Preventability of Medication Errors and ADEs in Ambulatory Care Older Patients.	Hernández et al. [27] 2018	2,218 older patients	Retrospective cohort study	High incidences of medication errors and adverse drug events were reported, with nearly half of adverse drug events considered preventable.
11	Impact of selected clinical pharmacy services on medication safety and prescription cost of patients attending a selected primary healthcare setting: a translational experience from a resource-limited country	Herath et al. [28] 2023	51 participants	Observational study	Clinical pharmacy interventions improved medication safety and significantly reduced prescription-related costs in a resource-limited setting.
12	Understanding the causes of preventable medication incidents for patients with mental illness in primary care	Ayre et al. [29] 2023	26 healthcare professionals	Qualitative study	Limited clinician knowledge regarding psychotropic medications and systemic healthcare pressures contributed substantially to medication incidents.
13	Identification of priorities for improvement of medication safety in primary care: a PRIORITIZE study	Car et al. [30] 2016	500 primary care clinicians	Cross-sectional study	Key priorities included improving medication reconciliation, discharge summaries, patient education, and communication between healthcare providers.

Quality of Included Studies

Among the included studies, six studies were of high quality, while six studies were moderate quality studies offering valuable insights despite some limitations, and one study (low quality) had small sample sizes and limited generalizability (Table 2). Overall, the studies provide valuable insights into medication safety issues, highlighting the importance of effective interventions in healthcare settings.

**Table 4:** Quality of the included studies *:* *Year of publication; RCTs: randomized controlled trials

No.	Title	Authors/Year*	Study Design	Quality
1	Professional, structural, and organizational interventions in primary care for reducing medication errors	Khalil et al. [18] 2017	Systematic review of RCTs	High
2	PRIME study: Prescription review to impede medication errors	Adil et al. [19] 2020	Prospective, observational study	Moderate
3	Economic analysis of the prevalence and clinical and economic burden of medication error in England	Elliot et al. [20] 2021	Observational, retrospective analysis	High
4	Medication errors in home care: a qualitative focus group study	Berland et al. [21] 2017	Qualitative focus group study	Moderate
5	Prevalence of adverse drug reactions in the primary care setting	Insani et al. [22] 2021	Systematic review and meta-analysis	High
6	Sources of unsafe primary care for older adults: a mixed-methods analysis of patient safety incidents	Cooper et al. [23] 2017	Cross-sectional, mixed-methods analysis	High
7	Medication safety challenges in primary care: Nurses’ perspective	Khalil et al. [24] 2018	Qualitative interview-based study	Low
8	Frequency of medication errors in primary care patients with polypharmacy	Koper et al. [25] 2013	Observational, cross-sectional study	Moderate
9	Medication incidents in primary care medicine: a prospective study	Gnädinger et al. [26] 2017	Prospective, observational surveillance study	Moderate
10	Incidence and Preventability of Medication Errors and ADEs in Ambulatory Care Older Patients	Hernández et al. [27] 2018	Retrospective, observational cohort study	High
11	Impact of selected clinical pharmacy services on medication safety	Herath et al. [28] 2023	Observational study	Moderate
12	Understanding the causes of preventable medication incidents for patients with mental illness	Ayre et al. [29] 2023	Qualitative study	High
13	Identification of priorities for improvement of medication safety	Car et al. [30] 2016	Cross-sectional study	Moderate

Prevalence/Incidence

Studies consistently underscore the high frequency of medication errors across various settings and populations. Adil et al. [19] identified 699 medication errors, with prescription errors being the most common (87.1%). Errors were more frequent in ICUs (52.8%) compared to general wards (42.8%), and adults (72%) had higher error rates than geriatric patients (18.8%). Gnädinger et al. [26] observed 2.07 incidents per year for general practitioners and 0.15 per year for pediatricians, with inappropriate dosing being the most frequent issue. In ambulatory care settings, high incidences of medication errors (12.5 events per 100 patient-years) were reported, with 42.9% of ADEs deemed preventable [27].

*Causes*****

The studies identified several underlying causes of medication errors. For instance, communication breakdowns between healthcare providers, lack of knowledge about medications, and unclear roles and responsibilities were commonly cited [21,24,29]. Medication errors were frequently associated with complex medication regimens in polypharmacy, where patients often took multiple medications, resulting in dosing errors and inappropriate prescriptions [25,27]. The elderly and patients with chronic conditions are disproportionately affected [25]. Mental health management posed additional risks, as primary care clinicians often lacked the expertise to prescribe psychotropic medications safely [29]. Systemic factors, such as inadequate discharge summaries and suboptimal reconciliation processes, were also prominent [30]. One study found that the majority of incidents were associated with a patient's inappropriate dose, while the second most frequent mistake was prescribing the wrong drug [26].

Impact

Medication errors remain a significant burden in primary care, with some studies estimating millions of errors annually [20]. ADRs, often preventable, affect up to 22.96% of primary care patients [22]. Older adults and patients with chronic conditions, such as diabetes and hypertension, were particularly vulnerable to ADEs [27]. These errors not only compromise patient safety but also incur substantial economic costs, as seen in England, where medication errors are estimated to cost the National Health Service (NHS) £98 million annually and contribute to over 1,700 deaths [20].

Prevention Strategies and Priorities

The studies emphasized several strategies for improving medication safety in primary care. Effective communication and standardized processes, such as discharge summary templates and medication reconciliation, were deemed critical [30]. Enhancing healthcare worker competence through education and training on medication management and safety protocols was a recurring theme [21,24]. Additionally, promoting a culture of open reporting and addressing systemic issues, such as workflow inefficiencies, were highlighted as key areas for intervention [23].

Effectiveness of Interventions

Interventions to mitigate medication errors yielded mixed results. Professional and organizational strategies in primary care showed limited success in reducing hospital admissions or mortality, with high variability in effectiveness across settings [18]. However, targeted interventions, such as prescription reviews by clinical pharmacists, significantly reduced prescription errors and enhanced patient safety [19]. In resource-limited settings, clinical pharmacy services demonstrated the dual benefit of improving medication safety and reducing prescription costs (Table 5) [28].

**Table 5:** Summary of the major characteristics and effectiveness of interventions to prevent medication errors RCTs: Randomized controlled trials

Intervention	Setting	Reported effectiveness
Professional, structural, and organizational interventions (30 RCTs)	Primary care	Limited or uncertain effect on hospital admissions, emergency visits, and mortality
Pharmacist-led prescription review (PRIME)	Prescriptions reviewed across wards including ICU and general wards	Significant reduction in the average number of errors per patient during follow-up
Clinical pharmacy services (medication review and counselling)	Primary healthcare, resource-limited setting	Improved medication safety and significantly reduced prescription-related costs
Clinician-identified priority interventions (medication reconciliation, discharge summaries, patient education, inter-provider communication)	Primary care	Priorities identified by 500 clinicians; effectiveness not directly evaluated

Discussion

Prevalence and Populations at Risk

The included studies in this systematic review indicate that medication errors remain highly prevalent across settings: prescription errors constituted the most common error type (87.1%) in the PRIME study by Adil et al. [19], and inappropriate dosing was the most frequent single incident type in primary care practice [26]. Previous research also found that nearly half of medication errors occurred in the primary care center, while about a quarter were patient medication errors [31]. In the included evidence, higher error frequencies were reported among adult patients and ICU prescriptions in the PRIME study [19] and among patients attended by general practitioners in the Swiss Sentinella surveillance network [26]. Similarly, previous studies noted that ICU environments, characterized by polypharmacy and complex care needs, amplify error risks [5,32]. In contrast, the lower frequency of errors reported by pediatricians may reflect differences in patient population, prescribing practices, or healthcare protocols [32]. Such disparities warrant further exploration to identify factors that contribute to safer practices in certain demographics. To mitigate this, robust prescription review systems integrated with automated alerts for potential errors have shown promise in reducing error rates [33]. Furthermore, adopting multidisciplinary reviews involving pharmacists could ensure a holistic evaluation of prescriptions, especially for vulnerable populations, and it has been shown to be effective [7,34]. The consistency in findings across these studies suggests that medication errors are not only prevalent but also contextually nuanced. Only one included study reported age-stratified rates: Adil et al. [19] found that geriatric patients accounted for a smaller proportion of errors (18.8%) than adults (72%). Whether this reflects more cautious prescribing in older patients is a hypothesis that cannot be confirmed from the included evidence and requires dedicated investigation. Supporting our findings, Samara et al. [35] also observed that elderly patients with polypharmacy often received potentially inappropriate medications, emphasizing the complex interplay of factors influencing error prevalence.

Causes of Medication Errors

The common causes of medication errors previously reported include systemic and human factors [36]. The most frequent prescription errors reported were omission of treatment duration, prescribing drugs by trade name instead of generic name, lack of adherence to guidelines, polypharmacy with potential drug interactions, duplicate therapy, and contraindicated drug combinations [37]. Similarly, our systematic review showed that communication breakdowns represent a recurrent issue. This aligns with the evidence that the main causes were prescribing errors in the primary care center and communication failures between patients and doctors for patient medication errors [1,31]. The fragmentation of healthcare delivery, particularly during patient transitions, exacerbates these problems, and incomplete reconciliation during handovers often leads to errors. Similar findings from a previous systematic review highlight that inappropriate prescribing, often due to inadequate reconciliation during transitions of care, exacerbates risks [38]. Park et al. [39] also reported that Emergency Department visits within a month of discharge are considerably increased by the frequency of inadvertent medication inconsistencies among critically sick older individuals during care transitions. Standardizing medication reconciliation protocols and integrating pharmacists into care teams have shown efficacy in reducing errors and improving outcomes for older adults [7,34]. Polypharmacy emerged as a critical driver of errors, particularly among elderly and chronically ill patients. This aligns with findings where chronic conditions such as diabetes and hypertension were commonly associated with ADEs. None of the included studies directly evaluated deprescribing interventions; nevertheless, deprescribing strategies tailored to the patient's condition and preferences represent a recommended approach, supported by external literature [34], to minimize unnecessary polypharmacy.

Clinical and Economic Impact

The impact of medication errors extends beyond individual patient safety, contributing to significant economic and societal burdens. The associated economic costs of millions annually for avoidable ADEs highlight the urgent need for effective interventions. This substantial economic burden of medication errors aligns with the global literature [40,41], further compounding the healthcare burden. These findings resonate with studies outside the review, which noted that enhanced pharmacovigilance could reduce preventable ADRs significantly [42,43]. Bates et al. [44] highlighted the high cost of ADEs, emphasizing preventability through better system designs. Digital health interventions, such as electronic health records with predictive analytics, have demonstrated cost-saving potential by reducing preventable ADEs [45]. The societal impact, including patient harm and mortality, is equally alarming, with one study by Elliot et al. [20] reporting over 1,700 deaths annually attributable to medication errors. This aligns with broader literature, which identified medical errors as one of the leading causes of death globally and the third cause of death in the USA [46,47]. Studies established that a quarter of the medication errors could cause severe harm, aligning with our findings [31]. These findings underscore the imperative to prioritize medication safety.

Prevention Strategies and Effectiveness of Interventions

Studies consistently emphasized the importance of systemic interventions to enhance medication safety. Standardized processes, such as discharge summary templates and medication reconciliation, were identified as critical. These recommendations align with WHO guidelines on transitions of care, which advocate for structured communication protocols to mitigate errors [48]. Healthcare teams can improve communication, utilize health information technology for decision-making, and foster a culture of safety by implementing standardized protocols for medication prescribing and dispensing [6,7].

Education and training for healthcare workers emerged as a recurrent theme, with a need to improve competence in medication management, particularly in complex settings like home care and primary care. This aligns with the findings of previous studies that demonstrated that targeted training programs significantly reduced prescribing errors [13,34,49]. Given the preventability of many ADRs, systematic medication reviews and patient education initiatives, as suggested by the WHO, could play pivotal roles in addressing this issue [50]. 

The review revealed mixed outcomes for interventions aimed at reducing medication errors. There was limited success with professional and organizational strategies, highlighting the variability in effectiveness across settings. However, prescription reviews by clinical pharmacists showed significant reductions in prescription errors. In resource-limited settings, clinical pharmacy services proved particularly impactful, and these interventions not only enhanced medication safety but also reduced prescription costs, demonstrating their dual benefit. This finding aligns with previous research that emphasized the cost-effectiveness of pharmacist-led interventions in low-resource environments [51,52]. Expanding the role of clinical pharmacists could bridge critical gaps in medication safety and costs resulting from medication errors.

Despite these successes, the review identified gaps in addressing systemic issues. For example, while education and training were frequently advocated, there is limited evidence on their sustained impact across settings. Addressing these systemic inefficiencies is crucial for sustaining improvements in medication safety. Unique challenges were observed in mental health management, where primary care clinicians often lacked expertise in prescribing psychotropic medications. This issue underscores the need for targeted education and systemic support to improve medication safety in this vulnerable population. Findings by Dilles et al. [53] highlighted the critical role of comprehensive training for home care nurses. Strategies like telehealth consultations, where nurses can access real-time guidance from pharmacists or physicians, could significantly enhance medication management in such settings [54]. We identified systemic and cultural barriers, such as unclear reporting mechanisms and inadequate training, as significant obstacles to medication safety. Previous research reported similar challenges stressing the importance of fostering a safety culture within healthcare organizations [55]. Introducing non-punitive error reporting systems, coupled with regular training on safety protocols, could address these challenges effectively. Promoting a culture of open reporting was another priority, with underreporting of medication incidents often obscuring the true extent of the problem. Encouraging transparent reporting and learning from errors is essential for fostering a safety-oriented culture. This perspective is supported by the concept of a “Just Culture” [56], which balances accountability with learning in healthcare systems. By enabling staff members to actively monitor the workplace and take part in safety initiatives, this concept enhances patient safety [57]. Though none of the included studies directly evaluated the adoption or effect of Just Culture in primary care, this concept supports our contextual recommendation derived from the broader patient safety literature [34,56,57] rather than a finding of this review.

Future Directions

This review highlights the need for a multifaceted approach to improving medication safety. In addition to existing strategies and persisting challenges, integrating advanced technologies such as artificial intelligence (AI) could offer novel solutions. None of the included studies evaluated AI-based interventions; however, external literature suggests that AI-driven tools for medication management, including automated prescription checks and real-time error detection, may hold promise in reducing errors [58]. Implementing such technologies could complement traditional interventions, particularly in high-risk settings like ICUs. Another emerging area is the role of patient engagement in medication safety. Encouraging patients to actively participate in their care through medication counseling and digital tools has been shown to reduce errors [59,60]. Leveraging patient-centered approaches could address gaps in communication and enhance adherence to treatment regimens. Finally, patients in low-resource settings or with limited health literacy often face higher risks of errors, and targeted interventions to these populations, as recommended by WHO’s “Medication Without Harm” initiative, could bridge these gaps and improve outcomes [61]. The WHO’s Medication Without Harm challenge seeks to reduce severe, avoidable medication-related harm by 50% globally over five years [61].

Clinical Implications

For primary care practitioners, the findings support routine structured medication review-particularly for older adults, patients with polypharmacy, and patients with mental illness-closer integration of clinical pharmacists into primary care teams, and systematic medication reconciliation at every care transition. For healthcare policymakers, the evidence supports investment in clinical pharmacy services, which improved safety and reduced prescription costs even in a resource-limited setting [28], standardized discharge and reconciliation protocols [30], and non-punitive incident reporting systems that enable learning from errors [23]. Because professional and organizational interventions alone showed limited or uncertain effectiveness [18], single-component interventions are unlikely to be sufficient, and multifaceted programs should be prioritized.

Strengths and Limitations

We acknowledge certain limitations for this review. One potential limitation is the exclusion of non-English studies, which may restrict the generalizability of the findings. Additionally, variability in study designs and outcome measures introduced heterogeneity, precluding meta-analysis. The reliance on published data might also omit valuable insights from unpublished studies or grey literature, and publication bias may therefore have inflated apparent error prevalence and intervention effects, although this could not be formally assessed in the absence of quantitative pooling. The restriction to English-language studies may likewise have excluded relevant evidence from non-English-speaking health systems, further limiting generalizability. In addition, the review protocol was not prospectively registered; several included studies encompassed mixed or adjacent care settings (hospital, home care, and surveillance networks) rather than exclusively primary care; and the inclusion of both primary studies and systematic reviews carries a residual risk of evidence overlap, despite the safeguards described in the methods.

Despite its limitations, this systematic review demonstrates several strengths. It employs a comprehensive search strategy across multiple databases and applies validated quality assessment tools to enhance methodological rigor and reduce potential bias. The use of validated quality assessment tools and adherence to PRISMA guidelines enhance the methodological rigor and reliability of the review’s findings. Including diverse study designs provides a nuanced understanding of the factors influencing medication errors in primary care.

## Conclusions

This systematic review emphasizes that medication errors remain significant global health concerns, particularly for vulnerable populations such as older adults, patients with polypharmacy, and individuals with mental illness. Medication errors are frequently associated with preventable adverse outcomes, underscoring the urgent need for targeted interventions. The findings suggest that professional, structural, and organizational interventions may have limited or inconsistent effects on reducing hospital admissions, emergency department visits, and mortality. However, clinical pharmacy interventions and improved medication management practices promise to reduce prescription errors, improve medication safety, and lower healthcare costs. It revealed systemic barriers to medication safety, including poor communication, lack of healthcare provider competence, inadequate reporting mechanisms, and unclear role definitions. These findings underscore the importance of addressing organizational culture, enhancing education and training, and fostering collaboration among healthcare professionals to mitigate medication safety risks. The complexity of medication safety issues highlights the need for context-specific strategies. Future research should focus on developing standardized protocols, communication, and training interventions for both healthcare providers and patients, leveraging technology to support clinical decision-making, and evaluating the long-term impacts of interventions. In particular, high-quality multicenter prospective studies are needed to evaluate technology-assisted medication safety interventions, including clinical decision support and AI-based error detection, in primary care. Additionally, prioritizing patient-centered approaches and fostering robust error-reporting cultures are essential for sustainable improvements in medication safety. By adopting these strategies, healthcare systems can move closer to the goal of safe and effective medication use for all patients.

## Appendices

**Table 6:** PubMed search strategy

Search Step	PubMed Search String
#1 Medication error concepts	("Medication Errors"[Mesh] OR "Medication Error*"[tiab] OR "Medication Errors"[tiab] OR "Medication Safety"[tiab] OR "Medication Safety"[Mesh] OR "Drug-Related Side Effects and Adverse Reactions"[Mesh] OR "Adverse Drug Event*"[tiab] OR "Adverse Drug Events"[tiab] OR "Adverse Drug Reaction*"[tiab] OR ADR[tiab] OR ADE[tiab] OR "Prescribing Errors"[tiab] OR "Prescribing Error*"[tiab] OR "Prescription Errors"[tiab] OR "Prescription Error*"[tiab] OR "Medication Incident*"[tiab] OR "Drug-Related Problem*"[tiab] OR DRP[tiab])
#2 Primary healthcare concepts	("Primary Health Care"[Mesh] OR "Primary Care"[tiab] OR "Primary Healthcare"[tiab] OR "Primary Health Care"[tiab] OR "General Practice"[Mesh] OR "General Practice"[tiab] OR "Family Practice"[Mesh] OR "Family Practice"[tiab] OR "Ambulatory Care"[Mesh] OR "Ambulatory Care"[tiab] OR "Outpatient Care"[Mesh] OR "Outpatient Care"[tiab] OR "Community Health Services"[Mesh] OR "Community Healthcare"[tiab] OR "Community Care"[tiab])
#3 Outcome concepts	("Patient Safety"[Mesh] OR "Patient Safety"[tiab] OR prevalence[tiab] OR incidence[tiab] OR epidemiology[tiab] OR causes[tiab] OR determinants[tiab] OR predictors[tiab] OR risk factors[tiab] OR prevention[tiab] OR intervention*[tiab] OR impact[tiab] OR burden[tiab] OR healthcare cost*[tiab] OR hospitali?ation*[tiab])
#4 Final search	#1 AND #2 AND #3
Limits applied	Publication dates: January 1, 2013–December 31, 2024; English language; Human studies

**Table 7:** Newcastle–Ottawa scale — cohort studies Domains: Selection (representativeness of exposed cohort; selection of non-exposed; ascertainment of exposure; outcome not present at start), Comparability (control for key confounders), Outcome (assessment of outcome; adequacy of follow-up length; adequacy of follow-up completeness).

Study (design)	Repr. exposed	Sel. non-exposed	Ascert. exposure	Outcome absent at start	Comparability	Outcome assessment	Follow-up long enough	Follow-up adequacy	Total stars	Overall
Hernández et al. 2018 [27] (retrospective cohort)	Low	Low	Low	Low	Moderate	Low	Low	Moderate	8/9	High

**Table 8:** Newcastle–Ottawa scale (cross-sectional adaptation) — cross-sectional/prevalence studies Domains: Selection (representativeness of sample; sample size justified; non-respondents described; ascertainment of exposure/measurement), Comparability (control for confounders), Outcome (assessment of outcome/method; statistical test appropriate).

Study	Repr. of sample	Sample size	Non-respondents	Ascertainment/measurement	Comparability	Outcome assessment	Statistics	Overall
Adil et al. 2020 [19] (prospective observational, prescription review)	Low	Moderate	Unclear	Low	Moderate	Low	Low	Moderate
Koper et al. 2013 [25] (cross-sectional, polypharmacy)	Moderate	Moderate	Unclear	Low	Moderate	Low	Moderate	Moderate
Gnädinger et al. 2017 [26] (prospective surveillance)	Low	Low	Moderate	Low	Moderate	Low	Low	Moderate
Cooper et al. 2017 [23] (cross-sectional mixed-methods)	Low	Low	Moderate	Low	Low	Low	Low	High
Car et al. 2016 [30] (cross-sectional PRIORITIZE)	Low	Low	Moderate	Moderate	Moderate	Low	Low	Moderate

**Table 9:** CASP qualitative checklist — qualitative studies Domains (Y = yes / criterion met; C = can’t tell; N = no): aims clear; qualitative method appropriate; design appropriate; recruitment appropriate; data collection appropriate; researcher–participant relationship considered (reflexivity); ethical issues addressed; rigorous analysis; clear findings; value/transferability.

Study	Aims	Method appr.	Design appr.	Recruit.	Data coll.	Reflexivity	Ethics	Rigorous analysis	Findings	Value	Overall
Berland et al. 2017 [21] (focus groups, home-care nurses)	Yes	Yes	Yes	Yes	Yes	Unclear	Yes	Yes	Yes	Yes	Moderate
Khalil et al. 2018 [24] (interviews, nurses)	Yes	Yes	Yes	Unclear	Yes	No	Unclear	Unclear	Yes	Yes	Low
Ayre et al. 2023 [29] (qualitative, mental-illness ME)	Yes	Yes	Yes	Yes	Yes	Unclear	Yes	Yes	Yes	Yes	High
